# Challenges and Support Strategies for Intensive Care Unit Nurses in the Organ Donation Process: A Scoping Review

**DOI:** 10.1111/jan.70384

**Published:** 2025-11-13

**Authors:** Nelson Selvaraj, Jessica Baillie, Jonathan Jones, Deborah Edwards

**Affiliations:** ^1^ School of Healthcare Sciences Cardiff University Cardiff UK; ^2^ Cwm Taf Morgannwg University Health Board Llantrisant UK; ^3^ University Library Service Cardiff University Cardiff UK; ^4^ Wales Centre for Evidence‐Based Care, School of Healthcare Sciences, Cardiff University Cardiff UK

**Keywords:** barriers, challenges, ICU nurse, intensive care unit, organ donation

## Abstract

**Aims:**

To provide an overview of the challenges that Intensive Care Unit nurses experience during the organ donation process and identify recommended support models or strategies that may assist them when caring for potential organ donors and their families during the organ donation process.

**Design:**

A scoping review was conducted in accordance with JBI methods.

**Data Sources:**

Cochrane Library, MEDLINE (via Ovid), Embase (via OVID), APA PsycINFO (via OVID), Scopus, OVID Emcare, Web of Science and CINAHL (via EBSCO) were searched from the first available start date of the individual database to December 2023.

**Review Methods:**

Eligible studies included peer‐reviewed empirical quantitative, qualitative and mixed method studies exploring the challenges experienced by Intensive Care Unit nurses during the organ donation process in adult intensive or critical care settings. Reviewers used Rayyan systematic review software to screen titles, abstracts and full‐text articles. Data were gathered using an adapted JBI data extraction tool for scoping reviews.

**Results:**

Twenty‐eight papers were included that were published between 1983 and 2023. Most studies (71.4%) used a qualitative approach. Seven key challenges were identified: direct patient care, care for the next of kin, concept of brainstem death, ethical challenges, emotional challenges, challenges around communication and organisational challenges. Several support models were identified including debriefing, training and education, and availability of local or national protocols and guidelines for organ donation.

**Conclusion:**

This scoping review provides an increased understanding of the challenges that Intensive Care Unit nurses experience during the organ donation process. Appropriate support models or strategies may potentially improve nurses' care experience during the organ donation process.

**Impact:**

Improved understanding of the nature of challenges during the organ donation process can facilitate the implementation of supportive strategies that may ultimately improve quality of care, consent rates and nurses' and donors' family experiences.

**Patient or Public Contribution:**

A public representative with family experience of organ donation was involved in developing the protocol and search strategy.

## 1 Introduction

Organ donation in Intensive Care Unit (ICU) settings is a complex process, and a successful donation process requires a multidisciplinary collaborative approach involving appropriate clinical and non‐clinical professionals (NHS Blood and Transplant 2021). ICU nurses have been extensively involved in the care of potential organ donors and their families for many decades (Simonsson et al. 2020). However, the donation process can challenge nurses' personal and professional beliefs (Emilie et al. 2022; Simonsson et al. 2020), and caring for a potential organ donor and their family can be a most demanding task for many ICU nurses (Emilie et al. 2022; Holthe and Husby 2023; Simonsson et al. 2020). Given the level of emotional stress and challenges that ICU nurses experience during the caring process of a potential donor, it is important that they are supported adequately during the organ donation process to improve their experiences and the overall outcome of the donation process.

### 1.1 Background

Organ donation and transplantation have attracted extensive international interest among experts and policy makers in the past two decades (Noyes et al. 2019). There are two main reasons for this: first, the global shortage of organs for transplantation, and secondly, the wide international variations in donation and transplantation activity (Global Observatory on Donation and Transplantation 2024). This has led to the development of strategic initiatives and prompted approaches to increase consent and transplantation rates (Olawade et al. 2025). In 2023, in the United States, the total number of patients transplanted was 132.1 patients per million population (pmp), compared to 69 pmp in the United Kingdom, 16.6 pmp in China and 1.0 pmp in Kenya (Global Observatory on Donation and Transplantation 2024). In the same year, there were 172,409 organs transplanted globally, meeting < 10% of global needs (Global Observatory on Donation and Transplantation 2024). The reasons for the global organ shortage are multifaceted (Madden et al. 2020). The critical shortage of viable organs means that patients must wait longer for their transplant operations, and unfortunately some patients die whilst on the organ waiting list (Olawade et al. 2025). The families of potential organ donors play a crucial role in determining whether organs will be donated for transplantation. Unfortunately, family refusal for organ donation remains a global issue and the reasons for refusal are complex and multifaceted (McLaughlin et al. 2025).

Organ donation is when an individual decides to donate an organ that is then transplanted to the body of a person with either a damaged, failed, or dysfunctional organ (Jawoniyi et al. 2018). There are two types of deceased organ donation practised worldwide (Madden et al. 2020); donation after brainstem death (DBD), which takes place after the diagnosis of death using specific neurological criteria, and donation after circulatory death (DCD), which takes place once death is diagnosed and confirmed using cardio‐respiratory criteria. The donation process begins when a potential organ donor is identified and includes several elements such as initiating a conversation with the patient's family to establish the patient's wishes on organ donation, performing at least two brainstem death tests, co‐ordinating with the local specialist organ donation team and supporting relatives throughout the donation process. Since most organs of deceased donors originate from patients being cared for in ICUs (Kotloff et al. 2015), nurses in these areas are in an ideal position to support families during end‐of‐life care decisions, initiate the organ donation discussion and refer potential organ donors to the specialist organ donation team. Caring for a potential organ donor and their family is, however, not a routine task for many ICU nurses (Simonsson et al. 2020). In addition, organ donation is a ‘time‐critical’ process, and once the decision has been made to donate the organs, the focus of care in the ICU changes dramatically from helping the patient overcome the critical illness to preserving their organs for potential donation (Emilie et al. 2022; Holthe and Husby 2023). The rapid change in the care process can challenge nurses' moral and ethical values and their views on what dignified care entails (YazdiMoghaddam et al. 2020).

Several studies suggest that ICU nurses experience tensions and conflicts between caring for a potential organ donor's bereaved family and society's need for more organs (Moghaddam et al. 2018; Simonsson et al. 2020; YazdiMoghaddam et al. 2020). Indeed the decision‐making process surrounding organ donation for donors' families can be extremely stressful (Simonsson et al. 2020), especially when the death of their loved one is unexpected and untimely, and therefore it is important that they are supported in a sensitive manner. The way in which the possibility of organ donation is presented to a grieving family can have a critical impact upon the decision that they make (McLaughlin et al. 2025). Evidence suggests that the presence of nurses when families are approached about the organ donation process may ease families' grief and thus facilitate the donor process (Flodén et al. 2011). However, approaching the donor family and initiating a conversation about possible organ donation with them in the ICU while simultaneously continuing to care for and support the donor patient, as well as the grieving family can be a most demanding and challenging task for many ICU nurses (Holthe and Husby 2023; Simonsson et al. 2020). A previous systematic review highlighted several challenges (YazdiMoghaddam et al. 2020) experienced by nurses around the organ donation process; however, the review had an exclusive focus on the DBD patients, meaning that the challenges around caring for DCD patients are not fully known. Given the array of approaches to organ donation (Global Observatory on Donation and Transplantation 2024) and the heterogenous nature of nursing care and responsibilities around the organ donation process (Flodén et al. 2011; Holthe and Husby 2023), a systematic scoping review to explore available evidence on the challenges that ICU nurses experience while caring for the potential organ donors (both DBD and DCD patients) is valuable. Identifying the areas of challenges that exist in the body of literature could facilitate the implementation of supportive strategies to ultimately improve the quality of care, consent rates and the overall experience of ICU nurses and donors' families. Additionally, the findings of this evidence synthesis would help researchers identify gaps in wider knowledge and the priorities for future research.

## 2 The Review

### 2.1 Aim

The primary aim of this scoping review was to explore the challenges experienced by ICU nurses while caring for patients during the organ donation process in the adult ICU settings. The secondary aim of this review was to identify recommended support models or strategies that may support ICU nurses in their role of caring for organ donors and their families during the organ donation process.

### 2.2 Design

This review was conducted in accordance with the JBI methodology for scoping reviews (Peters et al. 2020) and is reported according to the Preferred Reporting Items for Systematic Reviews and Meta‐Analyses extension for Scoping Reviews (PRISMA‐ScR) (see Data S1: PRISMA‐ScR Checklist Item) (Tricco et al. 2018). A scoping review was considered to be the most appropriate methodology to facilitate mapping of the complex and evolving challenges in caring for organ donors and their families, and the potential supportive strategies. A priori protocol was developed and registered on the Open Science Framework. The review was conducted in accordance with the protocol with no deviations. A public representative with family experience of organ donation was involved in developing the protocol and search strategy, which involved review and discussion of study documents. This ensured the focus of the review was relevant, and the search strategy included all appropriate search terms.

### 2.3 Search Method

A three‐step search strategy was carried out to identify published primary research evidence. A preliminary search of Medline (via OVID) and CINAHL (via EBSCO) was undertaken to identify appropriate papers on this topic. In the second stage, the text words contained in the titles and abstracts of relevant articles, and the index terms used to describe the articles were used to develop a full search strategy. A copy of MEDLINE (via Ovid) search is provided (see Data S2). Eight databases were searched with no date restrictions: CINAHL (via EBSCO), MEDLINE (via Ovid), Embase (via Ovid), APA PsycINFO (via Ovid), Scopus, OVID Emcare, Web of Science and Cochrane Library. The reference list of all included sources of evidence was screened for additional studies.

### 2.4 Inclusion and Exclusion Criteria

The inclusion and exclusion criteria of this review were based on the Participant, Concept, Context (PCC) framework (Peters et al. 2020). Table 1 provides an overview of the inclusion and exclusion criteria. Studies were eligible if they reported on challenges faced by ICU nurses (*participant*) in adult ICU settings during the organ donation process. Other health care professionals such as paediatric nurses, ICU physicians, Specialist Nurse Organ Donation (SNOD), student nurses and other allied healthcare professionals were excluded. The *concept* being explored was the ‘challenges’ experienced by ICU nurses while caring for patients and their families during the organ donation process. The terminology used to describe ‘challenges’ across studies varies, and it included ‘experiences’ (Emilie et al. 2022; Pearson et al. 2001) and ‘barriers’ (Holthe and Husby 2023). The *context* included adult ICU settings such as general intensive care units, including areas labelled as critical care units; or more specific units, such as medical or surgical ICUs, cardiothoracic ICUs, or neuro ICUs (British Association of Critical Care Nurses 2009). Studies that were conducted in paediatric/neonatal ICUs and non‐ICU settings such as medical and surgical wards, emergency departments, operating theatres and palliative care were excluded. Types of sources eligible for inclusion included quantitative, qualitative and mixed methods study designs. Non‐empirical evidence such as opinion papers, literature reviews, conference abstracts, editorials and discussion papers were excluded.

**TABLE 1:** Study identification.

PCC & Study design	Inclusion	Exclusion
Participant	ICU nurses in adult ICU settings	Paediatric nurses, ICU physicians, Specialist Nurse Organ Donation (SNOD), student nurses and other allied healthcare professionals
Concept	Challenges experienced by ICU nurses during the organ donation process	Living organ donation process
Context	Adult ICU/Critical Care settings—Medical or surgical ICUs, Cardiothoracic ICUs and Neuro ICUs	Paediatric or neonatal ICUs, Accident & Emergency, Operating theatres, Medical or Surgical wards and Palliative care settings
Study design	Qualitative, quantitative and mixed methods design	Opinion papers, literature review, conference abstracts, editorials, discussion papers and grey literature

### 2.5 Selection Process

Following the search, all identified citations were collated and imported into EndNote v.20 (Clarivate Analytics, PA, USA). Duplicates were removed before papers were imported into the Rayyan systematic review software package where they could be screened by the reviewers. Reviewers (N.S. and J.J.) undertook pilot testing by screening the title and abstract of the same 50 articles independently against the predetermined inclusion and exclusion criteria. Consensus was reached regarding the eligibility status of each of these articles with each article marked as ‘Yes’, ‘No’ or ‘Maybe’ and screening proceeded. There were no language restrictions at title and abstract screening; however, due to a lack of resources and funding to translate articles published in languages other than English, all potentially relevant non‐English studies identified at the full‐text stage were excluded. Articles that were categorised as ‘Yes’ and ‘Maybe’ were subject to the full‐text screening process and independently reviewed against the inclusion criteria by the two reviewers (N.S. and J.J.). Doubts and uncertainties at the title or full‐text screening were solved through discussion with a third reviewer (D.E.).

### 2.6 Quality Appraisal

Critical appraisal or risk of bias assessment is not a mandatory component of scoping reviews, given their exploratory nature and focus on mapping the breadth of available evidence (Peters et al. 2020). Therefore, a formal quality appraisal of the included studies was not conducted.

### 2.7 Data Extraction and Presentation

**FIGURE 1:**
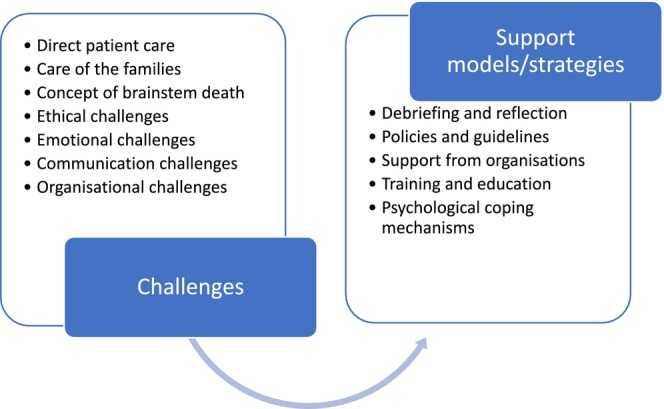
Scoping review findings.

An adapted version of the JBI data extraction tool was developed before the selection process and pilot tested on 10% of the final sample of included studies by two reviewers (N.S. and D.E.) to ensure reliability. The data extraction tool was modified iteratively throughout this process. Alterations included additional sections to facilitate the extraction of data relating to the type of organ donation (i.e., DCD or DBD) and years of ICU nurses' experience. This was to gain insight into whether these variables had any impact on the caring experiences of ICU nurses during the organ donation process. Data were extracted from included articles by one reviewer (N.S.) and a second reviewer (D.E.) checked for completeness and accuracy of the extracted data (Pollock et al. 2023). Disagreements were resolved through discussion with a third reviewer (J.B). Demographic data regarding the first author, year of publication, country, study aims, design and methods, sample size, age and years of ICU nurses' experience and type of organ donation were extracted and presented in a table accompanied by a narrative summary. Key findings relevant to the review questions were also extracted and presented narratively based on similarity in meaning accompanied by tables and figures. The challenges experienced by ICU nurses during the organ donation process were categorised as those relating to direct patient care, care of the families, concept of brainstem death, emotional challenges, ethical challenges, communication challenges and organisational challenges. The support models or strategies identified were grouped as follows: debriefing and reflection, training and education, guidelines and protocols, support from local unit and organisation, and psychological coping mechanisms (Figure 1).

## 3 Results

**FIGURE 2:**
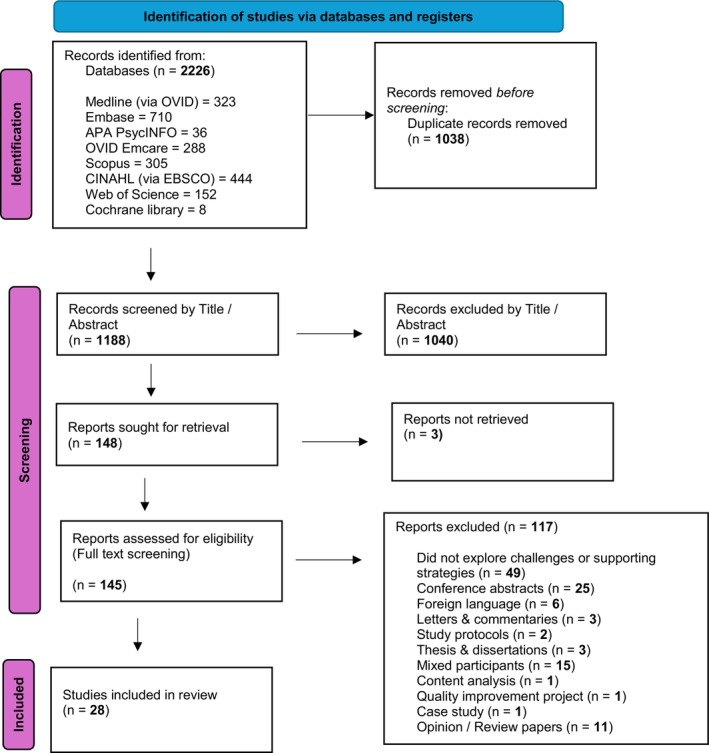
PRISMA flow diagram.

As shown in the PRISMA flow diagram (Figure 2), a search of eight databases yielded 2226 articles. After removing duplicates, 1118 articles remained and were uploaded to Rayyan for title and abstract screening. A total of 145 articles were subsequently included for full‐text review, of which 117 articles were excluded based on the eligibility criteria. In the end, 28 articles were included for the review. However, it is important to acknowledge that six non‐English studies were excluded which may have led to the exclusion of relevant literature published in other languages, thereby increasing the potential language bias in this scoping review.

### 3.1 Study Characteristics

A full list of included studies and their characteristics is included in Table 2. The articles were published between 1993 and 2023, and eight (28.5%) were published from 2020. Eight of the included papers were undertaken in European countries: UK (*n* = 2), Sweden (*n* = 4) and Norway (*n* = 2). The remaining sources comprised one article each from Australia, China, India, New Zealand, South Africa and Turkey; three articles each from Iran, Brazil, Canada and the United States; and two from Taiwan. Sample size ranged from five to 200, predominantly including female participants with a range of 0.5 to 34 years of experience in ICU.

**TABLE 2:** An overview of included studies.

Author/s, year, country and setting	Aims	Design and methods	Key findings (Challenges & Support models)
Atherton et al. (2020) New Zealand Intensive care unit	To explore the impact of simulation education on the nurses' perception and experiences of raising the option of tissue donation with families of deceased patients in an intensive care unit	Qualitative descriptive Semi‐structured interviews	Challenges in initiating conversations about organ donation with families. Simulation focussing on difficult conversation. Debriefing following simulation
Day (2001) United Staes of America Critical care unit	To describe the experiences of critical care nurses in making the shift from caring for a brain‐injured patient identified as a potential organ donor to maintaining a brain‐dead body	Interpretive phenomenological approach Semi‐structured interviews & observations	Lack of consensus around brainstem death diagnosis results in confusion related to care and treatment goals. Challenges in initiating conversations about organ donation with families
Deniz and Ayhan (2022) Turkey Intensive care unit	To compare the effectiveness of theoretical education and video‐assisted education in equipping ICU nurses to recognise brain death	Quantitative, randomisation Questionnaire	Video‐assisted training programme to improve knowledge level of organ donation care
Dodd‐McCue et al. (2005) United States of America General and neuro intensive care unit	To examine the impact of a protocol directed at increasing organ donation on the role stress and work attitudes of critical care nurses involved in potential organ donation cases	Quasi‐experimental prospective longitudinal study Surveys and interviews	Stressful caring process due to unclear communication of role responsibilities, competing role expectation and responsibilities and work overload The family communication co‐ordinator enhances effective communication among multidisciplinary team
Emilie et al. (2022) Sweden Intensive care unit	To examine ICU nurses' experiences of caring during the organ donor process from a caring science perspective.	Qualitative descriptive Semi‐structured interviews	Donor care was time‐consuming and energy‐intensive. Impact of close‐bond with donor families on emotional well‐being. Challenges in meeting the demands of in‐house routines during organ donation Support from the transplant coordinator and ICU physician, clear guidelines for donation process, reflection and debriefing following donation process, further education, inter‐professional simulations and/or workshop on organ donation process
Floden and Forsberg (2009) Sweden Intensive care unit	To describe intensive and critical care nurses' perceptions of organ donation based on their experience of caring for potential organ donors	Qualitative phenomenographic method Interviews	Donor care was challenging due to lack of knowledge and unfamiliar situation. Ethical challenges – Implementing medical interventions before the family had been informed about a possible brainstem death and organ donation. Ambiguity surrounding brainstem death Support from ICU physician throughout the donation process
Flodén et al. (2011) Sweden Intensive care unit	To examine ICU nurses' perceptions of their experiences of professional responsibilities and organisational aspects in relation to organ donation and how they understand and perceive brain death	Qualitative phenomenographic method Interviews	Understanding concept of brainstem death, lack of structure and guidelines on the organ donation process Support from ICU managers during the donation process, debriefing following donation process, having clear organisational structure around organ donation
Guido et al. (2009) Brazil Adult general intensive therapy unit	To examine stressors experienced by ICU nurses whilst delivering nursing care to potential organ donors	Qualitative descriptive Semi‐structured interviews	Exposure to unfamiliar nature of donor care, lack of communication among healthcare professionals, interacting with family members and supporting them during brainstem death Creating better working conditions and providing opportunities for nurses to reflect, discuss and learn from situations
He et al. (2020) China Intensive care unit	To explore factors that hinder ICU nurses from encouraging patients to donate organs	Qualitative Interviews	Heavy workload, lack of knowledge of the donation criteria and related policies and procedures, lack of confidence in supporting relatives due to lack of training and communication skills Training to improve nurses' awareness about organ donation and their willingness to participate in the co‐ordination of organ donation process Training to improve communication skills for organ donation
Hibbert (1995) Canada Neuro intensive care unit	To identify the stressors experienced by ICU nurses who care for organ donors and their families.	Retrospective explorative descriptive Semi‐structured interviews	Heavy workload and implementing complex and time‐consuming patient care, providing family members with relevant information and emotional support, informing family members about the diagnosis of brainstem death, approaching family members and raising the option of organ donation
Holthe and Husby (2023) Norway Intensive care unit	To describe the challenges faced by ICU nurses in the organ donation process	Qualitative descriptive Semi‐structured interviews	Practical challenges around direct patient care, challenging care for the next of kin, and ethical and emotional challenges Simulation training for the management of practical aspects of organ donor care, training to improve healthcare workers' communication skills, debriefing and follow‐ups after the care experience
Joshi et al. (2020) India Intensive care unit	To assess the effectiveness of a computer‐based self‐instruction module (CBSIM) in terms of improving the knowledge and acceptability of ICU nurses in relation to organ donation	Quantitative single arm study Pre and post‐test questionnaires	Computer‐based self‐instructional e‐learning modules improves ICU nurses' knowledge and acceptability of organ donation
Keshtkaran et al. (2015) Iran Intensive care unit	To understand the experiences of ICU nurses in care‐giving to the brain death of organ donor patients	Qualitative phenomenological Semi‐structured interviews	Ambiguity surrounding brainstem death diagnosis affecting the caring process and interaction with patient's relatives
Lemes and Bastos (2007) Brazil Intensive therapy unit	To understand ICU nurses' experience on the maintenance of potential organ donors	Qualitative ethnography Participative observation, interviews and documental analysis	Donor care was challenging due to lack of knowledge of the caring process. Emotional overload and internal conflict affecting quality care Training courses and lectures to acquire knowledge on brainstem death
Lin et al. (2010) Taiwan General and neuro intensive care units	The effect of training with regard to ICU nurses' knowledge, attitude, and motivation towards active participation to promote donation among deceased patients	Quantitative Pre and post‐test questionnaires	Training via lectures to improve nurses' knowledge of organ donation
Lin et al. (2014) Taiwan Intensive care unit	To explore the effects of an education program based on the Theory of Planned Behaviour (TPB) on ICU nurses' attitudes and behavioural intentions to advocate deceased organ donation	Randomised Pre and post‐test assessment	Educational training programme based on the Theory of Planned Behaviour to improve knowledge and enhance communication skills
Meyer and Bjork (2008) Norway Intensive care unit	To investigate the hospital‐based education in organ donation and explore challenges during organ donation process	Qualitative Semi‐structured interviews	Supporting family members while at the same time providing them with right amount of information, lack of formal local guidelines for organ donation Educational programme for newly qualified nurses, seminar on organ donation every alternative year for students and staff, providing time for nurses to reflect on their performance and learning, availabilities of practice guidelines for organ donation
Moghaddam et al. (2018) Iran Intensive care unit	To explore the nursing challenges of caring for patients diagnosed with brain death	Qualitative Semi‐structured interviews	Donor care was challenging, doubt surrounding brainstem death diagnosis affecting the caring process, informing family members about the diagnosis of brainstem death and on‐going communication with relatives, lack of support from local unit
Moraes et al. (2015) Brazil Intensive care unit	To understand the experiences and expectations of ICU nurses in caring for organ donors and their families	Qualitative phenomenological Interviews	Challenges in dealing with emotional needs of families due to lack of training Training programmes aimed at improving skills for breaking bad news
Pearson et al. (2001) Australia Critical care unit	To explore ICU nurses' experiences of caring for brain‐dead organ donor patients	Qualitative phenomenological approach Semi‐structured interview	Challenges in supporting families decision in organ support, ambiguity surrounding brainstem death, explaining brainstem death to family members
Pelletier‐Hibbert (1998) Canada Neuro intensive care unit	To identify different types of coping strategies used by ICU nurses to deal with the care of organ donors and their families	Qualitative Semi‐structured interviews	Challenges in maintaining potential organ donor on life‐sustaining treatment, supporting families during organ donation conversation Psychological coping mechanisms such as ‘depersonalisation’, ‘emotional distancing’, ‘maintaining normality’ and ‘positive reappraisal’. Peer support from colleagues, clinical nurse specialists and nursing management, protective time to reflect on experiences, appropriate educational preparation
Prins and Human (2019) South Africa Intensive care unit	To determine the knowledge and views of the ICU nurses regarding the early identification and referral of organ donors	Mixed method, experimental and exploratory Pre & post‐test questionnaire and interviews	Challenges in managing the potential organ donors on life‐sustaining treatment, dealing with the emotional needs of family members and informing them of the outcome of brainstem death. Poor communication from physicians and inadequate collaboration between physicians and nurses Having a national protocol or policy, in‐service training for every 6 months, debriefing following an organ donation process, having a clear local communication strategy
Ronayne (2009) United Kingdom General intensive care unit	To understand the experiences of ICU nurses with regard to brainstem death	Qualitative phenomenology Semi‐structured interviews	Experience of cognitive dissonance Supporting relatives and explaining the situation to relatives in a way that they understand the situation better
Salehi et al. (2013) Iran Intensive care unit	To describe the ICU nurses' experiences of care of brain‐dead donors in intensive care units.	Qualitative descriptive phenomenological approach Interviews	Stressful caring process and emotional challenges while supporting donor families
Simonsson et al. (2020) Sweden Intensive care unit	To explore ICU nurses with limited experience of caring for an organ donor during the donation process	Qualitative inductive approach Semi‐structured interviews	Donor care was challenging and emotionally draining, challenges in supporting families Education on donor care and collegial support, debriefing following donation process, easily accessible guidelines
Sophie et al. (1983) United Staes of America Intensive care unit	To understand ICU nurses' sentiments in relation to cadaver organ donation and their emotional reactions to caring for cadaver donors and their families	Mixed method Survey (quantitative & qualitative questions)	Donor care was heavy, emotionally demanding and draining Psychological coping mechanisms such as depersonalisation, interdisciplinary educational programme, having a clear hospital policies and protocols for organ donation, providing nurses with opportunities to reflect and ventilate their feelings
Starzomski et al. (2021) Canada Critical care unit	To describe the experiences of critical care nurses in the organ donation process	Qualitative Interviews and focus groups	Caring process was labour intensive and emotionally draining Education on brainstem death criteria for nurses, having evidence‐based protocol for organ donation formal training on organ donation, inclusion of pastoral care during organ donation process, debriefing post organ donation process, collegial support from nurse managers and multidisciplinary team
Watkinson (1995) United Kingdom Intensive care unit	To explore the knowledge, perceptions and attitudes of practising critical care nurses towards caring for brainstem dead cadaver organ donors and their families	Mixed method Survey and semi‐structured interviews	Experience of cognitive dissonance Peer support combined with formal/informal debriefing sessions, post donation care reflection and learning from experience

The included articles employed several different methodological approaches. Most used a qualitative approach (*n* = 20; 71.4%). The remainder were quantitative (*n* = 4; 14.2%), mixed methods (*n* = 4; 14.2%). Of the included papers, 25 (89.2%) explored the challenges experienced by ICU nurses while caring for patients during the organ donation process. Twenty‐two (78.5%) of the included papers identified several support models or strategies that may support ICU nurses in their role in caring for organ donors and their families during the organ donation process. Seventeen (60.7%) papers explored both challenges and support models or strategies.

### 3.2 Challenges Experienced by ICU Nurses During the Organ Donation Process

The articles included in this review identified seven challenges that ICU nurses experienced during the organ donation process: direct patient care, care of families, concept of brainstem death, emotional challenges, ethical challenges, communication challenges and organisational challenges.

#### 3.2.1 Direct Patient Care

The challenges associated with providing direct patient care during the organ donation process were reported by almost two‐thirds of the 28 included studies. Studies described the nature of direct patient care as physically demanding (He et al. 2020; Hibbert 1995; Sophie et al. 1983; Starzomski et al. 2021), complex (Hibbert 1995; Holthe and Husby 2023; Salehi et al. 2013; Simonsson et al. 2020) and time‐consuming (Emilie et al. 2022; Hibbert 1995). Dodd‐McCue et al. (2005) described direct patient care as a stressful process, especially when there was a lack of communication around role responsibilities and competing role expectations. This ultimately led to a delayed response about the treatment plan from the multidisciplinary team (Hibbert 1995). Heavy workload also led to a lack of time to communicate with patients' family members and obtain information about the organ donation decision of potential organ donors (He et al. 2020). The multifaceted nature of patient care was perceived to be stressful both by experienced and inexperienced nurses (Moghaddam et al. 2018; Salehi et al. 2013; Simonsson et al. 2020). Two studies discussed how sudden exposure to an unfamiliar situation of organ donor care increased the stress level among ICU nurses and impacted the quality of patient care (Floden and Forsberg 2009; Guido et al. 2009).

Three studies described challenges related to maintaining potential organ donors on life‐sustaining treatment (Holthe and Husby 2023; Pelletier‐Hibbert 1998; Prins and Human 2019). Lack of equipment (Guido et al. 2009), lack of knowledge of the caring process (Lemes and Bastos 2007; Salehi et al. 2013), and practical issues related to co‐ordination with the external shipment service (Holthe and Husby 2023) were further challenges affecting direct patient care.

#### 3.2.2 Care of the Families

The challenging nature of caring for the donors' families during the organ donation process was reported by 14 of the 28 included papers. Supporting donors' families was considered to be the most demanding and challenging aspect of the entire donation process (Holthe and Husby 2023; Moghaddam et al. 2018; Prins and Human 2019; Simonsson et al. 2020). Atherton et al. (2020), Day (2001), Guido et al. (2009) and Hibbert (1995) described that raising the option of organ donation with family members and supporting them during the donation process was a stressful and challenging prospect for many ICU nurses. Lack of confidence in supporting a donor's family due to limited experience (Simonsson et al. 2020) and lack of training and communication skills (He et al. 2020) were reported. This in turn affected ICU nurses' ability to provide family members with relevant information (Hibbert 1995; Meyer and Bjork 2008), explain the donation process to families in a way that they understood the enormity of the situation better (Ronayne 2009) and deal with the emotional needs of families (Moraes et al. 2015). Holthe and Husby (2023) reported a lack of relationship between the ICU nurse and donors' families as a huge barrier and this was seen as a challenge by many ICU nurses. Many felt that the ‘time‐critical’ nature of the donation process affected their ability to establish an effective therapeutic relationship with the donors' families (Holthe and Husby 2023) and provide compassionate care to them (Emilie et al. 2022).

#### 3.2.3 Concept of Brainstem Death

The diagnosis of brain death is complex and has an important role in the deceased organ donation process. However, a lack of understanding of and uncertainty about the concept and diagnosis of brainstem death among ICU nurses was highlighted by four studies (Flodén et al. 2011; Floden and Forsberg 2009; Holthe and Husby 2023; Moghaddam et al. 2018). Four studies discussed the impact of ICU nurses' lack of understanding of the concept of brainstem death on family interaction and the caring process (Day 2001; Keshtkaran et al. 2015; Moghaddam et al. 2018; Pearson et al. 2001). Donor families' perceptions of organ donation and brainstem death have also posed challenges for ICU nurses. Five studies found that it was challenging for nurses to inform families about the diagnosis of brainstem death especially when families had doubts or insufficient information about the concept of brainstem death (Guido et al. 2009; Hibbert 1995; Holthe and Husby 2023; Moghaddam et al. 2018; Prins and Human 2019).

#### 3.2.4 Emotional Challenges

Eleven studies explored ICU nurses' emotional challenges around caring for organ donors and their families. The overwhelming nature of the organ donation process was described by ICU nurses as emotionally demanding or draining (Sophie et al. 1983; Starzomski et al. 2021), challenging (Salehi et al. 2013) and energy‐consuming (Simonsson et al. 2020). Lemes and Bastos (2007) used the term ‘emotional overload’ to describe the experiences of nurses during the organ donation process. Two studies highlighted the emotional challenges faced by ICU nurses when they approached donors' relatives to explain the diagnosis of brainstem death despite their loved one seemingly looking alive (Pearson et al. 2001; Pelletier‐Hibbert 1998). Impressions of the donor corpse also caused emotional challenges (Holthe and Husby 2023). One study described the impact of a ‘close bond’ with donors' families during the donation process on ICU nurses' emotional well‐being (Emilie et al. 2022). Two studies (Ronayne 2009; Watkinson 1995) described the possible relationship between cognitive dissonance and emotional challenges.

#### 3.2.5 Ethical Challenges

Two studies explored the ethical challenges that ICU nurses experience while caring for potential donors and their families. Floden and Forsberg (2009) and Holthe and Husby (2023) found it was ethically challenging for ICU nurses to implement medical interventions (e.g., intra‐hospital transfer) before the family had been informed about a possible brainstem death and organ donation. Both studies described the ethical challenges that ICU nurses experienced when the concept of ‘saving lives’ was radically changed to ‘preserving organs’ for donation. Another situation perceived as a potential ethical conflict was when the organ donation process was prolonged by the wait for a matching recipient or due to uncertainty of brainstem death diagnosis (Holthe and Husby 2023).

#### 3.2.6 Communication Challenges

Four studies described challenges around communication during the organ donation process. Although ICU nurses play a vital role in facilitating various aspects of donor care, it was reported that they were not always involved when important information was communicated to donor families by ICU physicians. This made the subsequent communication with families extremely challenging for nurses, especially when doctors used medical jargon (Holthe and Husby 2023) or they did not know what was discussed with the family at the decision‐making meeting (Flodén et al. 2011; Holthe and Husby 2023). Poor communication from ICU physicians and inadequate collaboration between ICU physicians and nurses was identified by Prins and Human (2019), which in some cases led to difficulties in having honest communication with donor families. The study by Emilie et al. (2022) described how ICU nurses tried not to communicate verbally with the brain‐dead patients just in case that might confuse the family and lead them to doubt whether their loved one was alive.

#### 3.2.7 Organisational Challenges

Five of the 28 included studies reported on the various aspects of organisational challenges that influenced the organ donation process. Three studies (Emilie et al. 2022; Moghaddam et al. 2018; Simonsson et al. 2020) described challenges around meeting the demands of in‐house routines (e.g., informing the organ donation team and completing appropriate paperwork) during the organ donation process which was further complicated by a lack of support from the local unit or employer. Lack of structure or guidelines on the organ donation process was also highlighted by two studies (Flodén et al. 2011; Meyer and Bjork 2008).

### 3.3 Support Models or Strategies

Studies reported a variety of support models or strategies that may improve ICU nurses' experiences during the organ donation process. There were five categories of support models or strategies that emerged from the data, namely, debriefing and reflection, training and education, guidelines and protocols, support from the local unit and organization, and psychological coping mechanisms.

#### 3.3.1 Debriefing and Reflection

Eleven of the 28 studies highlighted the importance of debriefing following the donation process. Atherton et al. (2020) explored the impact of simulation education among ICU nurses to raise the option of donation emphasised the importance of debriefing following simulated activities and supporting participants to share their learning. Watkinson (1995) proposed peer support combined with formal debriefing sessions and learning from experience as a support strategy. Six other studies in this review also highlighted the importance of debriefing following the donation process (Emilie et al. 2022; Flodén et al. 2011; Holthe and Husby 2023; Prins and Human 2019; Simonsson et al. 2020; Starzomski et al. 2021). In addition to debriefing, five studies suggested post donation reflection as a strategy to facilitate open communication and peer learning (Emilie et al. 2022; Guido et al. 2009; Meyer and Bjork 2008; Pelletier‐Hibbert 1998; Watkinson 1995).

#### 3.3.2 Training and Education

Seventeen studies in this review suggested different forms of training and educational strategies. Five studies investigated the impact of various training and educational programmes on ICU nurses' confidence to raise donation (Atherton et al. 2020), knowledge about brainstem death identification and organ donation (Deniz and Ayhan 2022; Joshi et al. 2020; Lin et al. 2010) and attitudes towards organ donation (Lin et al. 2014). Of the five studies, one was a simulation training (Atherton et al. 2020); one used video illustrated lectures (Lin et al. 2010); one was an education programme based on the Theory of Planned Behaviour (Lin et al. 2014) and the remaining were delivered as video and computer‐based training respectively (Deniz and Ayhan 2022; Joshi et al. 2020). Three studies recommended simulation training as a support strategy for ICU nurses (Atherton et al. 2020; Emilie et al. 2022; Holthe and Husby 2023). Holthe and Husby (2023) proposed simulation training for the management of practical aspects of donor care, whereas Emilie et al. (2022) recommended inter‐professional simulations and workshops to enhance ICU nurses' confidence in organ donor care. Training and educating nurses on complex communication skills (He et al. 2020; Holthe and Husby 2023), breaking bad news (Moraes et al. 2015) and ethical aspects of donor care (Pelletier‐Hibbert 1998; Simonsson et al. 2020) were also proposed.

The studies reported a need for further education on the donor process (Emilie et al. 2022), brainstem death criteria (Lemes and Bastos 2007; Starzomski et al. 2021) and training to improve nurses' awareness about organ donation (He et al. 2020). Two studies recommended implementing appropriate support models for newly qualified or less experienced ICU nurses in the form of educational preparation and collegial support (Meyer and Bjork 2008; Simonsson et al. 2020). To improve nurses' knowledge of the organ donation process and donor care, more formal educational strategies in the form of seminars (Meyer and Bjork 2008), in‐service training programmes (Prins and Human 2019) and interdisciplinary education (Sophie et al. 1983) have been proposed.

#### 3.3.3 Guidelines and Protocols

A need for guidelines and protocols was highlighted in seven studies. There were some variations in terminology in the studies describing this source of support: ‘hospital policies’ (Sophie et al. 1983), ‘practice guidelines’ (Meyer and Bjork 2008), ‘organisational policy’ (Flodén et al. 2011) ‘protocol’ (Prins and Human 2019) and ‘clinical protocols/guidelines’ (Emilie et al. 2022; Simonsson et al. 2020; Starzomski et al. 2021). Sophie et al. (1983) proposed the importance of maintaining specific hospital policies regarding deceased organ donation in ICU settings. However, Simonsson et al. (2020) argue that having a hospital protocol or policy around organ donation may not always make a difference in donor care and emphasised the importance of making sure that these guidelines are easily accessible and written in a clear and understandable way. A practice guideline with clear details of responsibilities and organ donation procedures was proposed by Meyer and Bjork (2008) which may help improve nurses' confidence in approaching donors' families. Additionally, a national protocol or policy (Prins and Human 2019), protocols for evidence‐based donor management (Starzomski et al. 2021) and a clear organisational policy (Flodén et al. 2011) or guidelines (Emilie et al. 2022) for organ donation were some other suggestions offered that could help improve the organ donation process for ICU nurses.

#### 3.3.4 Support From Local Unit and Organisation

Almost half of the reviewed papers (*n* = 13) proposed various support strategies in relation to this that could help ICU nurses provide better quality donor care. The most commonly identified support strategy was support from colleagues during the donation process, including a senior colleague or peer (Pelletier‐Hibbert 1998; Simonsson et al. 2020; Watkinson 1995), transplant coordinator (Emilie et al. 2022; Pelletier‐Hibbert 1998), ICU ward manager (Flodén et al. 2011; Pelletier‐Hibbert 1998; Starzomski et al. 2021) and ICU physician (Emilie et al. 2022; Floden and Forsberg 2009). The importance of assigning one or two nurses exclusively for the donor patient and family during the organ donation process was proposed by two studies (Emilie et al. 2022; Meyer and Bjork 2008). Similarly, Pelletier‐Hibbert (1998) noted how working with the same group of nurses all the time helped some ICU nurses develop a better understanding of each other's needs. One paper proposed the implementation of a ‘family communication co‐ordinator’ protocol to clarify professional responsibilities and reduce role conflict in the organization (Dodd‐McCue et al. 2005). Two papers wrote of the necessity of having a clear local communication strategy (Prins and Human 2019) and of the importance of routine conversations with donors' families that were well planned together with the ICU physician in the local unit (Holthe and Husby 2023). Other strategies included ensuring new ICU nurses and professionals received an appropriate orientation to the local organ donation process (Sophie et al. 1983), providing better working conditions for ICU nurses as a way to minimise stress during the donation process (Guido et al. 2009) and including pastoral care workers and social care workers to support ICU nurses (Starzomski et al. 2021).

#### 3.3.5 Psychological Coping Mechanisms

Two studies discussed strategies related to psychological coping mechanisms that might enable ICU nurses to cope with the complex demands of the donation process. One paper described how the ‘depersonalisation’, a psychological mechanism helped some ICU nurses to cope with the stressful donor care (Sophie et al. 1983). Depersonalisation can be described as a mental response to persistent stress or trauma, which involves a profound feeling of detachment from one's emotional sense or being emotionally blunt (Medford 2012). Pelletier‐Hibbert (1998) described how ‘emotional distancing’ enabled some ICU nurses to carry out donor care without becoming emotionally overwhelmed. The role of ‘positive reappraisal’ as a coping mechanism was also highlighted in their study where nurses expressed a sense of pride and satisfaction when they witnessed positive donation outcomes. Other coping mechanisms such as ‘maintaining normality’ (Pelletier‐Hibbert 1998) were also suggested.

## 4 Discussion

The aim of this scoping review was to explore the challenges experienced by ICU nurses while caring for patients during the organ donation process in adult ICU settings and identify support models or strategies that may enable them to deliver optimal donor care. A broad range of international studies published over a 30‐year period (1983–2023) were included, and most studies originated from non‐European countries (*n* = 20). Of the 28 studies included, 10 papers focused on the care of DBD patients, four focused on the care of DCD patients and two focused on both DBD and DCD patients. However, the findings of the review indicated that the challenges experienced by ICU nurses did not vary significantly when caring for DBD and DCD patients. Moreover, it is important to note that 12 studies in this review did not specify the type of organ donation, warranting a degree of caution when interpreting the findings of this review. The following discussion explores challenges experienced by ICU nurses and support strategies, noting the interrelation of these.

### 4.1 Challenges Experienced by ICU Nurses During the Organ Donation Process

The review highlighted that ICU nurses encounter a range of challenges during the organ donation process. Some of these challenges arise from certain ‘nurse‐specific’ intrinsic factors such as lack of knowledge and skills of donor care, understanding the concept of brainstem death and communication skills. Other challenges are extrinsic in nature; for example heavy workload, lack of clear policies and guidelines on organ donation, lack of support from the local unit or employer and inadequate collaboration between ICU physicians and nurses. Challenges such as lack of knowledge about the care process and concept of brainstem death were previously identified by YazdiMoghaddam et al.'s (2020) review, which focused solely on DBD patients. In contrast, this review extends the scope by examining the challenges experienced by ICU nurses in caring for both DBD and DCD patients.

It is clear that donor care was not merely focused on donor patients, but encompassed positive working relationships with the donors' families, external agencies and the multidisciplinary team (Emilie et al. 2022; Holthe and Husby 2023). This implies that donor care requires a high level of competence from ICU nurses to create a caring atmosphere for people in their care and maintain professional relationships with colleagues, including those with contrasting perceptions and views about the organ donation process. Such a level of competence requires a combination of knowledge, skills and attitude (Nursing and Midwifery Council 2020), however, studies in this review indicated that ICU nurses lack knowledge and skills in various aspects of the organ donation process and that caring for potential donor patients and their families was complex. One possible explanation may be that donor care is not a routine aspect of ICU nursing practice and that a rapid shift in the focus of donor care from ‘saving life’ to ‘preserving organs’ for donation brought some unique challenges and exposed ICU nurses to an unfamiliar territory of donor care (Floden and Forsberg 2009; Guido et al. 2009). Interestingly, this was not unique to ICU nurses with varying levels of experience. A recent prospective multicentre study by Le Dorze et al. (2024) highlights similar challenges and tensions experienced by ICU physicians during the donation after circulatory death. This may be accentuated in settings lacking clear organisational structures, guidelines or support on the organ donation process, potentially leading to increased anxiety and psychological distress among ICU nurses (Flodén et al. 2011; Meyer and Bjork 2008). In the absence of formal supportive structures, nurses rely on previous experiences and support from colleagues to inform best practice (Flodén et al. 2011), and this was apparent in some other previous literature (Korsah and Schmollgruber 2023; Rafii et al. 2016).

Communication within the ICU is not merely limited to patients and their families, but also extends to team members where inter and intra‐professional communication is important (Olawade et al. 2025). Consistent with findings from a recent study (Le Dorze et al. 2024), this review identified that there was poor communication and collaboration between ICU physicians and nurses, which made the subsequent communication with families extremely challenging (Dodd‐McCue et al. 2005; Holthe and Husby 2023). Improved communication and effective collaboration among ICU healthcare professionals are important to facilitate a dignified death, as all professionals work towards the same goal (Ghattas and Abdou 2025).

A thorough understanding of the concept of brainstem death is crucial for ICU nurses when caring for patients who may be considered for donation after brainstem death; however it can challenge their personal beliefs on death (Coyle 2000). A lack of knowledge and understanding of the fundamental principles of brainstem death among ICU nurses contributed to ambiguity and uncertainty surrounding the diagnosis of brainstem death (Day 2001; Keshtkaran et al. 2015; Moghaddam et al. 2018; Pearson et al. 2001), but this needs to be further investigated, particularly in relation to cultural, societal and religious beliefs (Chen and LaBuzetta 2020). The appearance of the donor patient (warmth, colour and presence of vital signs) can complicate families' understanding of brainstem death and cause confusion. Such confusion could impact the quality of donor care and strengthen the denial of the patient's family (Keshtkaran et al. 2015). This finding resonates with the evidence from several studies that family members' understanding of the meaning of brainstem death is central in the organ donation decision‐making process (Lim et al. 2020; Ruta et al. 2021); thus improving their knowledge and understanding of brainstem death can increase the possibilities of consent for donation (Ruta et al. 2021).

The review identified that donor care was emotionally challenging for ICU nurses, and the findings of this review suggest that they were battling with their own feelings and emotions surrounding the organ donation process. Consistent with a systematic review (Danet Danet and Jimenez Cardoso 2019), a possible relationship between ‘cognitive dissonance’ and emotional challenges was uncovered by two studies in this review (Ronayne 2009; Watkinson 1995). Cognitive dissonance can be described as a psychological discomfort of holding two contradicting values or cognitions (Festinger 1957). This implies that despite possessing the knowledge of brainstem death, ICU nurses can be in dissonance with the appearance and warmth of the donor patient which is perceived to be emotionally challenging. From this, it is possible that the challenges expressed by other studies in this review (Pearson et al. 2001; Pelletier‐Hibbert 1998) were related to the concept of cognitive dissonance. This is important from grieving families' perspectives as they can also be in dissonance with the appearance of the donor patient (Gyllström Krekula et al. 2018), thus supporting them with timely and honest information without false hope is crucial. Further to this, there were ethical challenges that led ICU nurses to question their professional obligations (Floden and Forsberg 2009; Holthe and Husby 2023), but these were not unique to nurses. A qualitative study revealed how ICU consultants and other healthcare professionals struggled with their own conscience when life‐sustaining treatments were continued for the purposes of facilitating organ donation, with some experiencing tensions between organ donation in principle and the everyday practice of donation (Machin et al. 2022). Though included studies describe the ethical challenges nurses faced during donor care, information relating to how nurses addressed these challenges was not fully explored, highlighting the need for further investigation.

A successful donation depends on how the subject of organ donation is presented to donors' families (National Institute for Health and Care Excellence 2016). It was identified that raising organ donation conversations and supporting donors' families in decision‐making were considered to be the most challenging aspects of the entire donation process. The published guidelines offer various recommendations for best practice when communicating with families (National Institute for Health and Care Excellence 2016; Williment et al. 2023). Despite this, evidence suggests that communication is often perceived by family members as inadequate and sometimes inappropriate (Ma et al. 2021; Sque et al. 2018). It has been recommended that the donation conversation with families needs to be sensitive and timely (National Institute for Health and Care Excellence 2016), and there is a correlation between positive family experiences and subsequent consent to donate (Sque et al. 2018); however, literature suggests that there is no clear consensus on when is the best time to approach families regarding the donation (Meyer and Bjork 2008).

### 4.2 Support Models or Strategies

This scoping review identified several support models or strategies that may help ICU nurses to ensure donor care is optimal and that donors' families receive adequate support during the organ donation process. The strategies identified in this review were mostly broad claims and recommendations relating to debriefing and reflection, training and education, guidelines and protocols, support from the local unit and organisation and psychological coping mechanisms. However, the effectiveness of these strategies for supporting ICU nurses during the organ donation process is not fully known and therefore needs to be investigated further. Notably, some of the proposed support models or strategies are either related to conditions in the workplace or organisational culture. This highlights the importance of building a positive workforce culture towards organ donation, and calls for organisations to have supportive systems in place for ICU nurses and develop appropriate evidence‐based donor care policies to guide the delivery of high‐quality donor care (British Association of Critical Care Nurses 2009; Williment et al. 2023).

ICU nurses need appropriate training and education to be able to deliver effective donor care and support families during the organ donation process. This was further emphasised in a systematic review that reported that ICU nurses must have skills and knowledge that enable them to understand the concept and diagnosis of brainstem death, being well informed of the care process and how to interact with the families (YazdiMoghaddam et al. 2020), and these are similar to findings from this review. Despite this, there is currently a paucity of training and education for ICU nurses regarding the complex issues around organ donation (Atherton et al. 2020; Deniz and Ayhan 2022). Recognised training programmes such as the European Donor Hospital Education Programme may help ICU nurses gain necessary knowledge and skills required to manage the multidimensional nature of donor care effectively (Muthny et al. 2006). Other training strategies such as advanced communication skills training and preparation via simulation‐based learning to improve ICU nurses' confidence in various aspects of donor care (Atherton et al. 2020; Emilie et al. 2022; Holthe and Husby 2023) were also proposed. Nurses who have participated in an educational programme assign more value to the work accomplished and have a greater feeling of confidence, which increases the level of job satisfaction (Bryant and Parker 2020). Healthcare organisations should therefore consider the need for appropriate training strategies including simulation for ICU nurses to develop the specialised communication skills required during the organ donation conversation. Such strategies are now integrated in many best practice guidelines (L'her et al. 2020; National Institute for Health and Care Excellence 2016; NHS Blood and Transplant 2021). Evidence suggests that educating healthcare professionals on deceased donation appears to increase the rates of organ donation (Witjes et al. 2020), which could potentially reduce healthcare costs by improving resource utilisation (Olawade et al. 2025), however, studies in this review did not measure this outcome.

Given the extensive contact time that ICU nurses often have with family members during the organ donation process (Chen and LaBuzetta 2020), it is not surprising that they are often approached by family members and asked questions around brainstem death and institutional formalities. This review reveals that there is a lack of understanding about brainstem death among families. Abbasi et al. (2020) showed how lack of knowledge about brainstem death and fears and concerns among donor families negatively affected their organ donation decision‐making. Addressing families' concerns regarding brainstem death and donor care through robust ethical standards accurately and empathetically with confidence may enhance their acceptance of organ donation and influence public trust in donation positively (Chen and LaBuzetta 2020; Olawade et al. 2025).

The provision of adequate institutional support for ICU nurses through formal guidelines and protocols and collegial support is an essential requirement to optimise donor care. Evidence suggests that having clear organisational structures can minimise role conflicts and inconsistent practice (Olawade et al. 2025) and improve collaboration among the healthcare team (Ghattas and Abdou 2025), which can potentially improve patient outcomes (Xyrichis and Rose 2024). In units with clear organ donation protocols, nurses expressed a greater sense of protection and well‐being (Emilie et al. 2022). In addition to the structured protocols and guidelines, the Canadian study suggested post donation follow‐up for ICU nurses by appropriate external agencies and professionals (Starzomski et al. 2021). The support from the transplant coordinators (Specialist Nurses Organ Donation) also contributes immensely to ICU nurses' ability to cope with challenges associated with donor care (Emilie et al. 2022; Pelletier‐Hibbert 1998). Their role not only improves family experiences and collaborative team work (Noyes et al. 2019), but also has the potential to reduce the moral distress associated with donation that is experienced by nurses and donors' families (Madden et al. 2020; National Institute for Health and Care Excellence 2016). Further to this, creating a supportive environment for routine debriefing and reflection and encouraging nurses to share their learning facilitates open communication and peer learning (Garden et al. 2015); these are similar to findings from the review.

Healthcare organisations are responsible for creating and maintaining conducive learning environments for the workforce, and this is particularly important for newly qualified nurses (Nursing and Midwifery Council 2020). Evidence however suggests that the transition from higher education institutions to the workforce continues to be challenging for newly qualified nurses (Nour and Williams 2019; Smythe and Carter 2022). A previous study indicated that newly qualified nurses felt unprepared for the donor care process (Pelleriaux et al. 2008). When newly qualified nurses are faced with an unanticipated scenario such as donor care, it can increase feelings of insecurity and affect their performance (Smythe and Carter 2022). The combination of the complexity of patient care and lack of structured orientation can negatively impact newly qualified nurses' confidence and clinical performance (Ankers et al. 2018), potentially leading to feelings of pressure, burnout and a rise in attrition, which places a financial burden on the employer (Nour and Williams 2019; Smythe and Carter 2022). This suggests that these factors need to be carefully considered when planning preceptorship programmes for newly qualified nurses in critical care settings, so that they are prepared adequately for the realities of the organ donation process.

Finally, this review sheds light on psychological coping strategies which appear to help ICU nurses manage the emotional challenges endured during the donation process. The usefulness of ‘emotion‐focused’ psychological coping mechanisms, as highlighted in this review, such as ‘depersonalisation’ and ‘positive reappraisal’ (Pelletier‐Hibbert 1998; Sophie et al. 1983) in reducing the emotional stress on nurses has been explored positively in the past (Cruz et al. 2018). Although depersonalisation is seen as a natural psychological protective mechanism to overcome stressful events such as donor care, Alan et al. (2021) argue that it can occur as a result of burnout and potentially affect patient care, highlighting the importance of emotional support throughout the donation process. Thus, integrating psychological coping strategies in the current educational models and policies may help nurses to better cope with the emotional challenges and prepare a resilient workforce.

### 4.3 Limitations and Strengths

This scoping review followed a recognised framework rigorously and transparently. The search was undertaken systematically and with no limits applied to the date of publication. Furthermore, the involvement of a public representative in the development of the protocol and search strategy is a key strength of this review. This review is not without limits, however. The review was limited to primary research studies that were published only in English, which may have resulted in relevant studies or information being excluded. In keeping with the nature of a scoping review, the quality of the included studies was not appraised which may bias the conclusion of the review findings. Finally, the inclusion of studies that focused only on nurses in adult ICU settings may limit the generalisability of findings to other healthcare professionals working in acute and critical care settings.

## 5 Conclusion

The care of potential organ donors and their families falls within the scope of ICU nurses' responsibility but requires a high level of expertise. This review highlights that the donor care process is complex, challenging and demanding for ICU nurses. Many support models or strategies in this review refer to the need to improve ICU nurses' knowledge of the various aspects of donor care; however, this alone is not sufficient. They need targeted support from their peers, organisations and policy makers to be able to deliver effective donor care, meet families' needs and manage their own emotional well‐being. While the scoping review offers valuable insights into current support models or strategies, the effectiveness of these approaches was not evaluated in this scoping review. Therefore, further research is needed in this area to better understand the impact of these strategies on ICU nurses' experiences and organ donation consent rates across diverse clinical and cultural contexts. This may help policy makers and organisations to develop and implement evidence‐based support strategies to improve overall donor care and nurses' experiences.

## Author Contributions

All authors have agreed on the final version of the manuscript. N.S., J.B., D.E. contributed to the project concept and coordinated manuscript development and revision. N.S., J.B., D.E. were involved in drafting the manuscript or revising it critically for important intellectual content. N.S., J.B., D.E., J.J. developed the search strategy and J.J. conducted the database searches. N.S. and J.J. were involved in screening and study selection.

## Ethics Statement

The authors have nothing to report.

## Conflicts of Interest

The authors declare no conflicts of interest.

## Supporting information


**Data S1:** jan70384‐sup‐0001‐Supinfo01.docx.

**Data S2:** jan70384‐sup‐0002‐Supinfo02.docx.

## Data Availability

Data sharing not applicable to this article. As this is a scoping review, no datasets were generated or analyzed during the current study.

## References

[jan70384-bib-0001] Abbasi, P. , J. Yoosefi Lebni , P. Nouri , A. Ziapour , and A. Jalali . 2020. “The Obstacles to Organ Donation Following Brain Death in Iran: A Qualitative Study.” BMC Medical Ethics 21, no. 1: 83. 10.1186/s12910-020-00529-8. 32873305 PMC7466452

[jan70384-bib-0002] Alan, H. , F. E. Bacaksiz , A. K. Harmancı Seren , and H. A. Kurt . 2021. “Evaluating the Relationship Between Burnout Levels and Compassion Fatigue, Emotional Intelligence, and Communication Skills of Organ Transplant Coordinators.” Transplantation Proceedings 53, no. 2: 590–595. 10.1016/j.transproceed.2020.10.030. 33276958

[jan70384-bib-0003] Ankers, M. D. , C. A. Barton , and Y. K. Parry . 2018. “A Phenomenological Exploration of Graduate Nurse Transition to Professional Practice Within a Transition to Practice Program.” Collegian 25, no. 3: 319–325.

[jan70384-bib-0004] Atherton, S. , M. Crossan , and M. Honey . 2020. “The Impact of Simulation Education Amongst Nurses to Raise the Option of Tissue Donation in an Intensive Care Unit.” Nursing Praxis in Aotearoa New Zealand 36, no. 1: 20–29. 10.36951/27034542.2020.003.

[jan70384-bib-0005] British Association of Critical Care Nurses . 2009. “Standards for Nurse Staffing in Critical Care.” https://www.baccn.org/static/uploads/resources/BACCN_Staffing_Standards.pdf.

[jan70384-bib-0006] Bryant, S. , and K. Parker . 2020. “Participation in a Nurse Practitioner Fellowship to Instill Greater Confidence, Job Satisfaction, and Increased Job Retention.” Journal of the American Association of Nurse Practitioners 32, no. 10: 645–651. 10.1097/jxx.0000000000000313. 31651583

[jan70384-bib-0007] Chen, P. M. , and J. N. LaBuzetta . 2020. “A Qualitative Identification of Gaps in Understanding About Brain Death Among Trainees, Health Care Personnel and Families at an Academic Medical Center.” Neurohospitalist 10, no. 4: 266–271. 10.1177/1941874420923906. 32983344 PMC7495693

[jan70384-bib-0008] Coyle, M. A. 2000. “Meeting the Needs of the Family: The Role of the Specialist Nurse in the Management of Brain Death.” Intensive & Critical Care Nursing 16, no. 1: 45–50. 10.1054/iccn.1999.1469. 10790714

[jan70384-bib-0009] Cruz, J. P. , D. N. C. Cabrera , O. D. Hufana , N. Alquwez , and J. Almazan . 2018. “Optimism, Proactive Coping and Quality of Life Among Nurses: A Cross‐Sectional Study.” Journal of Clinical Nursing 27, no. 9–10: 2098–2108. 10.1111/jocn.14363. 29603804

[jan70384-bib-0010] Danet Danet, A. , and P. M. Jimenez Cardoso . 2019. “Emotional Experiences of Health Professionals in Organ Procurement and Transplantation. A Systematic Review.” Cirugía Española (English Edition) 97, no. 7: 364–376. 10.1016/j.cireng.2019.08.003. 30929746

[jan70384-bib-0011] Day, L. 2001. “How Nurses Shift From Care of a Brain‐Injured Patient to Maintenance of a Brain‐Dead Organ Donor.” American Journal of Critical Care 10, no. 5: 306–312. 11548563

[jan70384-bib-0012] Deniz, I. , and H. Ayhan . 2022. “The Effectiveness of Video Training in Improving Intensive Care Nurses' Knowledge About Brain Death Identification.” Nursing in Critical Care 29: 80–89. 10.1111/nicc.12863. 36414015

[jan70384-bib-0013] Dodd‐McCue, D. , A. Tartaglia , K. W. Veazey , and P. S. Streetman . 2005. “The Impact of Protocol on Nurses' Role Stress: A Longitudinal Perspective.” Journal of Nursing Administration 35, no. 4: 205–216. 15834260 10.1097/00005110-200504000-00010

[jan70384-bib-0014] Emilie, G. , K. Birgitta , B. Gunilla , et al. 2022. “Intensive Care Nurses' Experiences of Caring During the Organ Donor Process in Sweden—A Qualitative Study.” International Journal of Caring Sciences 15, no. 2: 720–726.

[jan70384-bib-0015] Festinger, L. 1957. A Theory of Cognitive Dissonance. Stanford University Press.

[jan70384-bib-0017] Floden, A. , and A. Forsberg . 2009. “A Phenomenographic Study of ICU‐Nurses' Perceptions of and Attitudes to Organ Donation and Care of Potential Donors.” Intensive & Critical Care Nursing 25, no. 6: 306–313. 10.1016/j.iccn.2009.06.002. 19608419

[jan70384-bib-0016] Flodén, A. , M. Berg , and A. Forsberg . 2011. “ICU Nurses' Perceptions of Responsibilities and Organisation in Relation to Organ Donation‐A Phenomenographic Study.” Intensive & Critical Care Nursing 27, no. 6: 305–316. 10.1016/j.iccn.2011.08.002. 21872472

[jan70384-bib-0018] Garden, A. , D. Le Fevre , H. Waddington , and J. Wells . 2015. “Debriefing After Simulation‐Based Non‐Technical Skill Training in Healthcare: A Systematic Review of Effective Practice.” Anaesthesia and Intensive Care 43: 300–308. 10.1177/0310057X1504300303. 25943601

[jan70384-bib-0019] Ghattas, A. H. S. , and H. A. Abdou . 2025. “Challenges and Best Practices for Moving Forward in Interprofessional Collaboration in Critical Care Units: Nurses' Perspectives.” BMC Nursing 24, no. 1: 317. 10.1186/s12912-025-02860-0. 40133871 PMC11934773

[jan70384-bib-0020] Global Observatory on Donation and Transplantation . 2024. “International Figures on Donation and Transplantation.” https://www.transplant‐observatory.org/newsletter‐transplant‐2024/.

[jan70384-bib-0021] Guido, L. d. A. , G. F. d. C. Linch , R. Andolhe , C. C. Conegatto , and C. C. Tonini . 2009. “Stressors in the Nursing Care Delivered to Potential Organ Donors.” Revista Latino‐Americana de Enfermagem 17, no. 6: 1023–1029. 20126946 10.1590/s0104-11692009000600015

[jan70384-bib-0022] Gyllström Krekula, L. , U. Forinder , and A. Tibell . 2018. “What Do People Agree to When Stating Willingness to Donate? On the Medical Interventions Enabling Organ Donation After Death.” PLoS One 13, no. 8: e0202544. 30142168 10.1371/journal.pone.0202544 PMC6108459

[jan70384-bib-0023] He, D. , L. Lei , L. Yue , Z. Dongmei , and L. Yu . 2020. “A Qualitative Analysis of the Influencing Factors About ICU Nurses in Chongqing Refusing to Donate Their Organs.” Chinese Journal of Practical Nursing 36, no. 3: 210–214. 10.3760/cma.j.issn.1672-7088.2020.03.010.

[jan70384-bib-0024] Hibbert, M. 1995. “Stressors Experienced by Nurses While Caring for Organ Donors and Their Families.” Heart & Lung 24, no. 5: 399–407. 8567305 10.1016/s0147-9563(05)80062-7

[jan70384-bib-0025] Holthe, E. , and V. S. Husby . 2023. “Barriers to Organ Donation: A Qualitative Study of Intensive Care Nurses' Experiences.” Dimensions of Critical Care Nursing 42, no. 5: 277–285. 10.1097/DCC.0000000000000596. 37523727

[jan70384-bib-0026] Jawoniyi, O. , K. Gormley , E. McGleenan , and H. R. Noble . 2018. “Organ Donation and Transplantation: Awareness and Roles of Healthcare Professionals—A Systematic Literature Review.” Journal of Clinical Nursing 27, no. 5–6: e726–e738. 10.1111/jocn.14154. 29098739

[jan70384-bib-0027] Joshi, P. , M. Thomas , G. Lakshmanan , et al. 2020. “Pilot Testing of a Computer‐Based Self‐Instructional Module on Organ Donation for Improvement in Knowledge and Acceptability of Nurses Working in the Intensive Care Unit of a Tertiary Care Institute.” Indian Journal of Physiology and Pharmacology 64, no. 5: S76–S81. 10.25259/IJPP_284_2020.

[jan70384-bib-0028] Keshtkaran, Z. , F. Sharif , E. Navab , and S. Gholamzadeh . 2015. “Lived Experiences of Iranian Nurses Caring for Brain Death Organ Donor Patients: Caring as ‘Halo of Ambiguity and Doubt’.” Global Journal of Health Science 8, no. 7: 281–292. 10.5539/gjhs.v8n7p281. PMC4965685 26925919

[jan70384-bib-0029] Korsah, E. K. , and S. Schmollgruber . 2023. “Barriers and Facilitators to End‐Of‐Life Care in the Adult Intensive Care Unit: A Scoping Review.” International Journal of Africa Nursing Sciences 19: 100636. 10.1016/j.ijans.2023.100636.

[jan70384-bib-0030] Kotloff, R. M. , S. Blosser , G. J. Fulda , et al. 2015. “Management of the Potential Organ Donor in the ICU: Society of Critical Care Medicine/American College of Chest Physicians/Association of Organ Procurement Organizations Consensus Statement.” Critical Care Medicine 43, no. 6: 1291–1325. 10.1097/ccm.0000000000000958. 25978154

[jan70384-bib-0031] Le Dorze, M. , R. Barthélémy , O. Lesieur , et al. 2024. “Tensions Between End‐Of‐Life Care and Organ Donation in Controlled Donation After Circulatory Death: ICU Healthcare Professionals Experiences.” BMC Medical Ethics 25, no. 1: 110. 10.1186/s12910-024-01093-1. 39385217 PMC11462860

[jan70384-bib-0032] Lemes, M. M. D. , and M. A. R. Bastos . 2007. “The Maintenance Care of Potential Organ Donors: Ethnographic Study on the Experience of a Nursing Team.” Revista Latino‐Americana de Enfermagem 15, no. 5: 986–991. 10.1590/s0104-11692007000500016. 18157452

[jan70384-bib-0033] L'her, E. , T. Geeraerts , J.‐P. Desclefs , et al. 2020. “Simulation‐Based Teaching in Critical Care, Anaesthesia and Emergency Medicine.” Anaesthesia, Critical Care & Pain Medicine 39: 311–326. 10.1016/j.accpm.2020.03.010 32223994

[jan70384-bib-0034] Lim, K. J. , T. T. J. Cheng , M. S. Jeffree , et al. 2020. “Factors Influencing Attitude Toward Organ and Tissue Donation Among Patients in Primary Clinic, Sabah, Malaysia.” Transplantation Proceedings. 10.1016/j.transproceed.2020.01.007 32146022

[jan70384-bib-0035] Lin, L. M. , C. C. Lin , C. L. Chen , and C. C. Lin . 2014. “Effects of an Education Program on Intensive Care Unit Nurses' Attitudes and Behavioral Intentions to Advocate Deceased Donor Organ Donation.” Transplantation Proceedings 46, no. 4: 1036–1040. 10.1016/j.transproceed.2013.12.039. 24815121

[jan70384-bib-0036] Lin, L. M. , C. C. Lin , H. D. Lam , and C. L. Chen . 2010. “Increasing the Participation of Intensive Care Unit Nurses to Promote Deceased Donor Organ Donation.” Transplantation Proceedings 42, no. 3: 716–718. 10.1016/j.transproceed.2010.03.022. 20430155

[jan70384-bib-0037] Ma, J. , L. Zeng , T. Li , X. Tian , and L. Wang . 2021. “Experiences of Families Following Organ Donation Consent: A Qualitative Systematic Review.” Transplantation Proceedings 53: 501–512. 33483168 10.1016/j.transproceed.2020.09.016

[jan70384-bib-0038] Machin, L. L. , J. Cooper , H. Dixon , and M. Wilkinson . 2022. “Organ Donation in Principle and in Practice: Tensions and Healthcare Professionals' Troubled Consciences.” BioSocieties 17, no. 3: 347–367. 10.1057/s41292-020-00219-z.

[jan70384-bib-0039] Madden, S. , D. Collett , P. Walton , et al. 2020. “The Effect on Consent Rates for Deceased Organ Donation in Wales After the Introduction of an Opt‐Out System.” Anaesthesia 75, no. 9: 1146–1152. 10.1111/anae.15055. 32372409 PMC7496553

[jan70384-bib-0040] McLaughlin, L. , N. Mays , M. Al‐Haboubi , et al. 2025. “Potential Donor Family Behaviours, Experiences and Decisions Following Implementation of the Organ Donation (Deemed Consent) Act 2019 in England: A Qualitative Study.” Intensive & Critical Care Nursing 86: 103816. 10.1016/j.iccn.2024.103816. 39217721

[jan70384-bib-0041] Medford, N. 2012. “Emotion and the Unreal Self: Depersonalization Disorder and de‐Affectualization.” Emotion Review 4, no. 2: 139–144.

[jan70384-bib-0042] Meyer, K. , and I. T. Bjork . 2008. “Change of Focus: From Intensive Care Towards Organ Donation.” Transplant International: Official Journal of the European Society for Organ Transplantation 21, no. 2: 133–139. 17944801 10.1111/j.1432-2277.2007.00583.x

[jan70384-bib-0043] Moghaddam, H. Y. , Z. S. Manzari , A. Heydari , E. Mohammadi , and I. Khaleghi . 2018. “The Nursing Challenges of Caring for Brain‐Dead Patients: A Qualitative Study.” Nursing and Midwifery Studies 7, no. 3: 116–121. 10.4103/nms.nms_14_17.

[jan70384-bib-0044] Moraes, E. L. d. , F. F. Neves , M. J. D. Santos , M. A. B. Merighi , and M. C. K. B. Massarollo . 2015. “Experiences and Expectations of Nurses in Caring for Organ Donors and Their Families.” Revista da Escola de Enfermagem da USP 49: 129–135. 10.1590/S0080-623420150000800018. 26959164

[jan70384-bib-0045] Muthny, F. A. , S. Wiedebusch , G. A. Blok , and J. van Dalen . 2006. “Training for Doctors and Nurses to Deal With Bereaved Relatives After a Sudden Death: Evaluation of the European Donor Hospital Education Programme (EDHEP) in Germany.” Transplantation Proceedings 38, no. 9: 2751–2755. 10.1016/j.transproceed.2006.08.155. 17112822

[jan70384-bib-0046] National Institute for Health and Care Excellence . 2016. Organ Donation for Transplantation: Improving Donor Identification and Consent Rates for Deceased Organ Donation. https://www.nice.org.uk/Guidance/CG135. 32091685

[jan70384-bib-0047] NHS Blood and Transplant . 2021. “Organ Donation and Transplantation 2030: Meeting the Need A Ten‐Year Vision for Organ Donation and Transplantation in the United Kingdom.” https://www.odt.nhs.uk/odt‐structures‐and‐standards/key‐strategies/meeting‐the‐need‐2030/.

[jan70384-bib-0048] Nour, V. , and A. M. Williams . 2019. ““Theory Becoming Alive”: The Learning Transition Process of Newly Graduated Nurses in Canada.” Canadian Journal of Nursing Research 51, no. 1: 6–13. 10.1177/0844562118771832 29768951

[jan70384-bib-0049] Noyes, J. , L. Mclaughlin , K. Morgan , et al. 2019. “Process Evaluation of Specialist Nurse Implementation of a Soft Opt‐Out Organ Donation System in Wales.” BMC Health Services Research 19, no. 1: 414. 31234832 10.1186/s12913-019-4266-z PMC6591913

[jan70384-bib-0050] Nursing and Midwifery Council . 2020. “Principles of Preceptorship.” https://www.nmc.org.uk/globalassets/sitedocuments/nmc‐publications/nmc‐principles‐for‐preceptorship‐a5.pdf.

[jan70384-bib-0051] Olawade, D. B. , S. Marinze , N. Qureshi , K. Weerasinghe , and J. Teke . 2025. “Transforming Organ Donation and Transplantation: Strategies for Increasing Donor Participation and System Efficiency.” European Journal of Internal Medicine 133: 14–24. 10.1016/j.ejim.2024.11.010. 39572291

[jan70384-bib-0052] Pearson, A. , S. Robertson‐Malt , K. Walsh , and M. Fitzgerald . 2001. “Intensive Care Nurses' Experiences of Caring for Brain Dead Organ Donor Patients.” Journal of Clinical Nursing 10, no. 1: 132–139. 11820230 10.1046/j.1365-2702.2001.00447.x

[jan70384-bib-0053] Pelleriaux, B. , L. Roels , D. Van Deynse , J. Smits , O. Cornu , and C. Delloye . 2008. “An Analysis of Critical Care Staff's Attitudes to Donation in a Country With Presumed‐Consent Legislation.” Progress in Transplantation 18, no. 3: 173–178. 10.1177/152692480801800305. 18831482

[jan70384-bib-0054] Pelletier‐Hibbert, M. 1998. “Coping Strategies Used by Nurses to Deal With the Care of Organ Donors and Their Families.” Heart & Lung 27, no. 4: 230–237. 9713714 10.1016/s0147-9563(98)90034-6

[jan70384-bib-0055] Peters, M. D. J. , C. Marnie , A. C. Tricco , et al. 2020. “Updated Methodological Guidance for the Conduct of Scoping Reviews.” JBI Evidence Synthesis 18, no. 10: 2119–2126. 10.11124/jbies-20-00167. 33038124

[jan70384-bib-0056] Pollock, D. , M. D. J. Peters , H. Khalil , et al. 2023. “Recommendations for the Extraction, Analysis, and Presentation of Results in Scoping Reviews.” JBI Evidence Synthesis 21, no. 3: 520–532. 10.11124/jbies-22-00123. 36081365

[jan70384-bib-0057] Prins, L. , and L. Human . 2019. “Early Identification and Referral of Organ Donors in Five Private Hospitals: A Survey to Determine the Knowledge and Views of Critical Care Professional Nurses Pre and Post a PowerPoint Training Intervention.” Southern African Journal of Critical Care: The Official Journal of the Critical Care Society 35, no. 2: 48. 10.7196/SAJCC.2019.v35i2.370. PMC10103843 37063498

[jan70384-bib-0058] Rafii, F. , A. Nikbakht Nasrabadi , and M. A. Karim . 2016. “End‐of‐Life Care Provision: Experiences of Intensive Care Nurses in Iraq.” Nursing in Critical Care 21, no. 2: 105–112. 10.1111/nicc.12219. 26487503

[jan70384-bib-0059] Ronayne, C. 2009. “A Phenomenological Study to Understand the Experiences of Nurses With Regard to Brainstem Death.” Intensive & Critical Care Nursing 25, no. 2: 90–98. 10.1016/j.iccn.2008.06.001. 18657425

[jan70384-bib-0060] Ruta, F. , G. Gallo , P. Ferrara , et al. 2021. “Translating Knowledge About Organ and Tissue Donation Using Webinars: An Exploratory Study in Italy.” Transplantation Proceedings 53: 1792–1797. 34275598 10.1016/j.transproceed.2021.06.018

[jan70384-bib-0061] Salehi, S. , T. Kanani , and H. Abedi . 2013. “Iranian Nurses' Experiences of Brain Dead Donors Care in Intensive Care Units: A Phenomenological Study.” Iranian Journal of Nursing and Midwifery Research 18, no. 6: 475–482. 24554946 PMC3917131

[jan70384-bib-0062] Simonsson, J. , K. Keijzer , T. Sodereld , and A. Forsberg . 2020. “Intensive Critical Care Nurses' With Limited Experience: Experiences of Caring for an Organ Donor During the Donation Process.” Journal of Clinical Nursing 29, no. 9: 1614–1622. 10.1111/jocn.15195. 31971283

[jan70384-bib-0063] Smythe, A. , and V. Carter . 2022. “The Experiences and Perceptions of Newly Qualified Nurses in the UK: An Integrative Literature Review.” Nurse Education in Practice 62: 103338. 10.1016/j.nepr.2022.103338. 35462212

[jan70384-bib-0064] Sophie, L. R. , J. C. Salloway , G. Sorock , P. Volek , and F. K. Merkel . 1983. “Intensive Care Nurses' Perceptions of Cadaver Organ Procurement.” Heart & Lung: The Journal of Critical Care 12, no. 3: 261–267. 6551374

[jan70384-bib-0065] Sque, M. , W. Walker , T. Long‐Sutehall , M. Morgan , G. Randhawa , and A. Rodney . 2018. “Bereaved Donor Families' Experiences of Organ and Tissue Donation, and Perceived Influences on Their Decision Making.” Journal of Critical Care 45: 82–89. 29413728 10.1016/j.jcrc.2018.01.002

[jan70384-bib-0066] Starzomski, R. , A. E. Molzahn , R. McCarthy , B. Budz , and S. Matheson . 2021. “Organ Donation: A Cross‐Canada Perspective of Critical Care Nursing Practice.” Canadian Journal of Critical Care Nursing 32, no. 4: 21–30. 10.5737/23688653-3242130.

[jan70384-bib-0067] Tricco, A. C. , E. Lillie , W. Zarin , et al. 2018. “PRISMA Extension for Scoping Reviews (PRISMA‐ScR): Checklist and Explanation.” Annals of Internal Medicine 169, no. 7: 467–473. 10.7326/M18-0850. 30178033

[jan70384-bib-0068] Watkinson, G. E. 1995. “A Study of the Perception and Experiences of Critical Care Nurses in Caring for Potential and Actual Organ Donors: Implications for Nurse Education.” Journal of Advanced Nursing 22, no. 5: 929–940. 8568068 10.1111/j.1365-2648.1995.tb02645.x

[jan70384-bib-0069] Williment, C. , L. Beaulieu , A. Clarkson , et al. 2023. “Organ Donation Organization Architecture: Recommendations From an International Consensus Forum.” Transplantation Direct 9, no. 5: e1440. 10.1097/txd.0000000000001440. 37138552 PMC10150918

[jan70384-bib-0070] Witjes, M. , N. E. Jansen , J. van Dongen , et al. 2020. “Appointing Nurses Trained in Organ Donation to Improve Family Consent Rates.” Nursing in Critical Care 25, no. 5: 299–304. 10.1111/nicc.12462. 31294520 PMC7507830

[jan70384-bib-0071] Xyrichis, A. , and L. Rose . 2024. “Interprofessional Collaboration in the Intensive Care Unit: Power Sharing Is Key (But Are We Up to It?).” Intensive & Critical Care Nursing 80: 103536. 10.1016/j.iccn.2023.103536. 37783179

[jan70384-bib-0072] YazdiMoghaddam, H. , Z.‐S. Manzari , and E. Mohammadi . 2020. “Nurses' Challenges in Caring for an Organ Donor Brain Dead Patient and Their Solution Strategies: A Systematic Review.” Iranian Journal of Nursing and Midwifery Research 25, no. 4: 265–272. 10.4103/ijnmr.IJNMR_226_18. 33014736 PMC7494161

