# Supplementary material for: Challenges and Support Strategies for Intensive Care Unit Nurses in the Organ Donation Process: A Scoping Review
Source: J Adv Nurs. 2025 Nov 13;82(8):7717–34. doi: 10.1111/jan.70384 (PMC13356446; doi:10.1111/jan.70384)
Supplement: Supplementary file 2 — Data S2: jan70384‐sup‐0002‐Supinfo02.docx. [file JAN-82-7717-s001.docx]

**Supplementary File S2: Database: Ovid MEDLINE(R) ALL <1946 to December 08, 2023>**

| **#** | **Query** | **Results to 8 Dec 2023** |
| --- | --- | --- |
| 1 | exp "Tissue and Organ Procurement"/ | 26421 |
| 2 | exp Organ Transplantation/ | 239417 |
| 3 | exp Tissue Donors/ | 84429 |
| 4 | exp Tissue Transplantation/ | 204450 |
| 5 | ((organ or organs or tissue) adj6 (donor* or donat* or transplant*)).mp. | 136858 |
| 6 | (transplant adj6 (donor* or donat*)).mp. | 16235 |
| 7 | (brain adj2 death).mp. | 13647 |
| 8 | (brain adj2 dead).mp. | 2898 |
| 9 | circulatory death.mp. | 1795 |
| 10 | 1 or 2 or 3 or 4 or 5 or 6 or 7 or 8 or 9 | 474785 |
| 11 | exp Critical Care Nursing/ | 2629 |
| 12 | ((critical care or intensive care or acute care or ICU or ccu) adj5 nurs*).mp. | 16620 |
| 13 | 11 or 12 | 16620 |
| 14 | 10 and 13 | 323 |
